# Maze Procedures for Atrial Fibrillation, From History to Practice

**DOI:** 10.4021/cr79w

**Published:** 2011-09-20

**Authors:** Charles Kik, Ad J.J.C. Bogers

**Affiliations:** a Department of Cardiothoracic surgery, Thoraxcentre, Erasmus Medical Centre, The Netherlands

**Keywords:** Surgical treatment, Atrial fibrillation, Maze procedure

## Abstract

Atrial fibrillation may result in significant symptoms, (systemic) thrombo-embolism, as well as tachycardia-induced cardiomyopathy with cardiac failure, and consequently be associated with significant morbidity and mortality. Nowadays symptomatic atrial fibrillation can be treated with catheter-based ablation, surgical ablation or hybrid approaches. In this setting a fairly large number of surgical approaches and procedures are described and being practised. It should be clear that the Cox-maze procedure resulted from building up evidence and experience in different steps, while some of the present surgical approaches and techniques are being based only on technical feasibility with limited experience, rather than on a process of consequent methodology. Some of the issues still under debate are whether or not the maze procedure can be limited to the left atrium or even to isolation of the pulmonary veins or that bi-atrial procedures are indicated, whether or not cardiopulmonary bypass is to be applied and which route of exposure facilitates an optimal result. In addition, maze procedures are not procedures guide by electrophysiological mapping. At least in theory not in all patients all lesions of the maze procedures are necessary. A history and aspects of current practise in surgical treatment of atrial fibrillation is presented.

## Introduction

As one of the most common cardiac arrhythmias, atrial fibrillation is being present in up to 2% of the general population and in approximately 10% of patients over the age 60. The actual incidence and prevalence of atrial fibrillation may be substantially higher, due to undetected asymptomatic atrial fibrillation and underdetection in patients with paroxysmal atrial fibrillation. Because the numbers are increasing, it is realistic to state that atrial fibrillation is a growing epidemic [1, 2].

Atrial fibrillation is often considered to be a mild arrhythmia. However, atrial fibrillation may result in significant symptoms, (systemic) thrombo-embolism and tachycardia-induced cardiomyopathy leading to a diminished quality of life with increased morbidity and mortality [3]. Symptoms, thrombo-embolism and cardiomyopathy are all an indication for intervention. The increased risk for thrombo-embolism is the main indication for intervention in this regard. While pharmaco-medical treatment for atrial fibrillation is aimed at control of rate or rhythm [4], invasive treatment for atrial fibrillation is aimed at rhythm control. An invasive approach may consist of percutaneous catheter treatment, surgery or a hybrid approach. This review concentrates on surgical maze procedures.

## Definitions

The classification system published jointly by the American Heart Association, American College of Cardiology and European Society of Cardiology is the most widely used system to classify atrial fibrillation [5]. Atrial fibrillation is defined as either paroxysmal, persistent, or permanent. When a patient has had two or more episodes, atrial fibrillation is considered recurrent. If recurrent atrial fibrillation terminates by itself, it is defined paroxysmal; but if it does not, it is defined persistent. Termination by pharmacologic therapy or electrical cardioversion before expected spontaneous termination does not change the designation of paroxysmal. Permanent atrial fibrillation includes cases of long-standing atrial fibrillation (>1 year), in which cardioversion has not been indicated or has failed to convert the arrhythmia. This terminology applies to episodes of atrial fibrillation that last more than 30 seconds and that are unrelated to a reversible cause.

In 2003 Cox has suggested a new system, defining intermittent and continuous atrial fibrillation [6]. In this system, the patient is either in atrial fibrillation all the time (continuous) or not (intermittent). This has not been widely accepted by professional organizations.

## History of Maze Surgery for Atrial Fibrillation

The early developments in surgery for atrial fibrillation were characterized by a stepwise approach; unfortunately some of the more recent surgical procedures are being applied on the basis of technical feasibility with only limited experience.

### Left atrial isolation

In 1980, a left atrial isolation procedure was described, that proved to confine atrial fibrillation to the left atrium [3]. This procedure allowed a synchronized sinus rhythm to the right atrium and both of the ventricles.

Interestingly from the point of view of cardiac failure, left atrial isolation resulted in normal cardiac hemodynamics in patients with normal left ventricular function. Apparently the left atrium functioned as a passive conduit, through which the normalized right-sided cardiac output was passed to the left ventricle. However, the risk for systemic thrombo-embolism was unaffected because the left atrium stayed in fibrillation. Further steps in maze surgery for atrial fibrillation ideally needed to address abolishing of atrial fibrillation, re-establishing of sinus rhythm, maintaining atrio-ventricular synchrony, restoring normal atrial transport function, and eliminating the risk of thrombo-embolic events.

### Cox-maze procedure

The clinical application of the Cox-maze procedure resulted from animal studies [7-9]. The animal experiments lead to the suggestion that a mechanism for atrial fibrillation could be found in a macro re-entrant circuit around the orifice of the left atrial appendage and the ostia of the four pulmonary veins [8].

Based on these findings the procedure was designed to consist of transsection of the atria, with a dorsal incision from the tricuspid valve annulus to the mitral valve annulus. It became clear that clinical electrophysiological mapping, in relation to atrial anatomy, in atrial fibrillation would improve to understand the mechanisms resulting in atrial fibrillation [10, 11]. Both in atrial flutter and atrial fibrillation three components could be identified: firstly macro re-entrant circuit(s), secondly passive atrial conduction in the atrium outside the macro re-entrant circuit(s), and thirdly atrio-ventricular conduction. With the typical electrophysiological patterns of these components most of the atrial arrhythmias could be explained. The next step was to develop a surgical procedure to interrupt all macro re-entrant circuits. As a result the sinus node should be able to function normally. This procedure was first applied clinically in 1987 [9-12].

This Cox-maze I procedure was associated at follow up with left atrial dysfunction and with the inability to accelerate to an appropriate sinus tachycardia in relation to exercise. For these reasons, the procedure was further developed into the Cox-maze III procedure [12-15].

### Cox-maze III

The operative procedure known as the Cox-maze III operation resulted in a higher recovery of sinus rhythm, showed better sinus node function, needed less often implantation of a permanent pacemaker, and showed improved transport function. In addition, the Cox-maze III procedure was technically somewhat less demanding than earlier procedures [16-19], but median sternotomy and use of cardio-pulmonary bypass were necessary [13, 20]. The Cox-maze III procedure showed to be effective in treating atrial fibrillation [16-20].

Unfortunately the incidence of morbidity associated with the procedure was not negligible. Re-exploration for postoperative blood loss was an important factor and the necessity of implantation of a permanent occurred in approximately 10 % of the patient. Because of these circumstances and results, only a limited number of surgeons incorporated the procedure in their practise. Definitely there was a need for less invasive and simpler approaches to the surgical treatment of atrial fibrillation.

### Maze modifications

Both basic research and clinical practise resulted in a better understanding of the electrophysiology of atrial fibrillation and the relevant clinical pathophysiology in this regard. An important factor was the attempt to develop practical systems for clinical mapping. In 1998 important findings with regard to arrhythmogenic-foci-originating atrial fibrillation were described [21]. In patients with paroxysmal atrial fibrillation the pulmonary veins were found to be a critical area of triggers for start of paroxysmal atrial fibrillation. More importantly these areas of triggers were assumed to be accessible for successful ablation with radiofrequency methods. These results lead to a new surgical approach for pulmonary vein isolation. Although the importance of the pulmonary veins in the pathophysiology of atrial fibrillation was clear, some patients demonstrated a different and much more complex pattern of starting and ongoing of the rhythm disturbance. Therefore, the therapy in those patients goes beyond simple isolation of the pulmonary vein [22, 23].

Some fifteen years ago the surgical cryomaze procedure was clinically introduced. The procedure consisted mainly of application of cryoablation lines, with the idea of replacing the surgical incisions. In 1999, the first full Cox-maze procedure was performed using exclusively cryothermal ablation instead of the classical cutting and sewing technique. Shortly thereafter the Cox-maze III operation was further developed into the so-called Cox-maze IV procedure. The Cox-maze IV procedure included bilateral isolation of the pulmonary veins with a connecting lesion between the two encircling lesions. This connecting lesion replaced the previously applied box lesion. This modification was based on the findings of the studies into arrhythmogenic-foci-originating atrial fibrillation [21]. The cryosurgical maze operation was also applied as a minimally invasive procedure with an approach through a right antero-lateral thoracotomy [24]. A number of the following operative modifications to the previous maze procedures were introduced along new surgical devices and various technologies for ablation. These new devices and technologies allowed further development of surgical procedures and protocols for treating atrial fibrillation. At present, the concept of surgery for atrial fibrillation is to follow the pattern of the surgical incisions with ablative lines. In this regard a number of devices have been introduced clinically, using different sources of energy, such as unipolar and bipolar radiofrequency [25, 26], microwave [27], laser [28], cryo-ablation [29], and high-frequency ultrasound [30]. The goal of the original surgical procedures as well as all the new techniques was to create lines of transmural ablation in order to block conduction in circuits of re-entry. Nevertheless, follow up demonstrated that not all of these ablative techniques resulted in transmural lesions [31, 32]. Practically however, it was demonstrated that the maze procedure with cutting and sewing could be replaced by a maze procedure consisting of a more sophisticated technique that is technically less demanding. Furthermore these new techniques can be performed with a less invasive or minimally invasive approach.

## Developments

At present a fairly large number of surgical approaches and procedures are described and being practised [3]. It should be clear that the Cox-maze procedure resulted from building up evidence and experience in different steps, while some of the present surgical approaches and techniques are being based only on technical feasibility with limited experience, rather than on a process of consequent methodology [3]. Some of the issues still under debate are whether or not the maze procedure can be limited to the left atrium or even to isolation of the pulmonary veins or that bi-atrial procedures are indicated, whether or not cardiopulmonary bypass is to be applied and which route of exposure facilitates an optimal result.

## Factors Affecting the Results of Surgical Maze Procedures for Atrial Fibrillation

In the analysis of the results from follow up of maze procedures some factors were found that were negatively associated with success of the maze procedure. In this regard the duration of the arrhythmia before the maze procedure turned out to be associated with failure. An explanation may be found in a relevant adverse remodelling of atrial myocardium with more extensive tissue fibrosis. Consequently, differences in initiation and conduction of action potentials as well as increasing dimensions of the left atrium develop. This increased size of the left atrium is in itself also an important risk factor for recurrence of atrial fibrillation, with a cut-off size of the dimensions of the left atrium of 6 cm [33-36]. Patients with an increased age at the time of surgery are also exposed to an increased risk of failure of the maze procedure, but this finding is not confirmed in all series [33, 34]. Furthermore, the type of atrial fibrillation, structural heart disease and rheumatic heart disease, have all been pointed at to limit the success of maze procedures, but could not be uniformly confirmed [3].

Some of the issues in patients undergoing surgical ablation procedures are similar to those that were encountered when the Cox-maze procedure was first developed [3]. In this regard improvement in classification and selection of patients for a certain procedure may provide further improve to the outcome of the modified maze procedures. Before applying such a maze procedure, some basic characteristics of both the patient as well as the atrial arrhythmia should be documented. This concerns the presence of any other organic heart disease; the type and duration in time of atrial fibrillation; the size of both atria; the presence of clots in the left atrial appendage or the presence of a patent foramen ovale, limiting the ability to perform a procedure without cardiopulmonary bypass; whether the procedure is intended for curing atrial fibrillation or is using cardiopulmonary bypass for an other indication.

At present the general opinion is that only a limited number of patients with lone atrial fibrillation will ever be offered to undergo the classical open-heart maze procedures [3]. However, there are an increasing number of surgeons nowadays who apply ablation for atrial fibrillation. These ablation procedures are performed with devices and, in comparison with the Cox-maze procedure are simpler easier to do. Therefore, the issue is not so much the risk-benefit ratio of the surgical Cox-maze procedure anymore, but should be concentrated on selection using with evidence-based decision trees regarding specific maze procedures for specific clinical matters. In this regard this selection concerns at least the specific maze procedure, the timing of the maze procedure, the type of equipment and the energy source. There is a need for ongoing development to further diminish the invasiveness and to simplify the surgical approach in the surgical treatment of atrial fibrillation. However, innovations in surgical maze procedures should not compromise the results [3].

## Current Surgical Strategies

Different aspects of surgery for atrial fibrillation are at present a matter of discussion. Electrophysiological mechanisms are still an important subject for research. Mapping studies are being undertaken in this regard. The surgical approach should meet the needs of the individual of the patient. This is especially important when atrial fibrillation is being treated as concomitant disease. Minimally invasive surgery is getting more and more attention. These techniques are feasible, but long-term follow up is not completely established yet. All of these aspects should have their impact on surgical strategy.

### Mechanism

Although it was previously thought that atrial fibrillation was maintained by multiple macro-reentrant circuits, there is evidence that focal triggers can be responsible for the initiation of paroxysmal atrial fibrillation as well [21, 37]. Therefore, preoperative electrophysiological diagnostic studies into areas of early activation, may allow surgeons to identify the particular triggers of atrial fibrillation in individual patients. Unfortunately, the analysis of multiple atrial electrocardiograms over long periods of time has been difficult. Thus, it usually is not possible to locate the precise focal point of origin responsible for the initiation or maintenance of atrial fibrillation in the majority of patients.

### Surgical approach

Nevertheless, present day surgery for atrial fibrillation asks for a custom-made approach. Different patients with different characteristics of their disease are best treated with an individualised approach [3]. Currently, surgery through a median sternotomy is applied in patients in whom atrial fibrillation in operated along with surgery for other structural heart disease. This approach is also being applied in patients in whom atrial fibrillation is intended to be treated with an isolated maze operation and who have an increased risk for a less invasive or minimally invasive approach. In general, a complete biatrial maze lesion set is indicated in these patients. At present the surgical cut-and-sew Cox-maze procedure is abandoned unless intra-operative ablation devices are not at hand. In this regard it is important that the surgeon is trained in the specific device that is being applied and that the surgeon is aware of both the possibilities and the limitations of the device that is being used.

Although nowadays the risks of cardiopulmonary bypass are limited, it is being used to a minimum. Both cardiopulmonary bypass time as well as aortic cross-clamp time are being kept to the minimum that is required to serve the purpose of the operation. This is equally relevant with regard to the surgical treatment of atrial fibrillation. In maze surgery parts of the procedure can be performed off bypass. When using cardiopulmonary bypass parts of the surgical maze procedure can be done before or after aortic cross-clamping. When using purse-string sutures in the right atrial wall to introduce the ablation device, most of the right atrial ablation lines can be facilitated without extracorporeal circulation. Should a right atriotomy be performed for treating other structural heart disease, the right-sided lesions can be made easily using the atriotomy.

With regard to left atrial ablation lines, both encircling of all pulmonary veins as well as encircling of the right and left pulmonary veins from the epicardial side can be performed off-pump. If necessary or if indicated this can off course also be performed with cardiopulmonary bypass. Using extra-corporeal circulation, encircling of all pulmonary veins as an endocardial approach, may be indicated in redo surgery in which adhesions limit the pericardial accessibility and the pulmonary veins are not easily reachable. Opening of the left atrium facilitates application ablation to create connecting lesion and a mitral valve isthmus lesion to include the coronary sinus.

### Minimally invasive surgery

Although the ongoing development to further diminish the invasiveness of the surgical approach in the treatment of atrial fibrillation is a relevant issue, innovative steps in surgical maze procedures should not compromise the results [3]. In this regard evidence is being build up for the minimally invasive surgical approaches. An isolated surgical maze procedure can be performed through a minimally invasive entry using a right antero-lateral mini-thoracotomy or port-entries for video-assisted surgery. This exposure can of course also be used when surgical ablation is to be combined with other procedures using this approach.

This exposure can also be an alternative after previous cardiac surgery, when median resternotomy would present an increased risk [3]. In this setting extra-corporeal circulation is instituted using femoral arterial entry and femoral venous return. As described above, the right atrial ablation lines can be applied off-pump or on-pump. On cardio-pulmonary bypass and after cross-clamping the aorta, the left atrium is opened with a longitudinal incision in the inter-atrial groove. This left atriotomy is used to apply all the additional left-sided ablation lines. Completion of the encircling of the right and left pulmonary veins with a connecting line or completion of a box lesion around all pulmonary veins is performed. The maze is completed with a connecting lesion to the left atrial appendage and with a connecting lesion to the mitral valve isthmus. The left atrial appendage can be resected with oversewing from the epicardial side, but increasingly the orifice of the left atrial appendage is oversewn from the endocardial side, leaving the remnants of the appendage. Surgery on the mitral valve is best performed after completion of the maze procedure to ensure an adequate ablation of the left atrial isthmus. In the specific setting of surgery on the tricuspid valve and of repair of an atrial septal defect, extracorporeal circulation requires double venous cannulation and the ablation procedures can be applied through the right atrial incision.

As an extension of the clinical experience, operative procedures applying only pulmonary vein isolation with or without resection or oversewing of the left atrial appendage are nowadays being performed. The advantage of these procedures is that it can be performed without the use of cardiopulmonary bypass. The evidence of these procedures is building up, and further evaluation is being awaited. The practical advantage of these procedures is that they can be done as a totally endoscopic procedure, necessitating only a limited exposure and limited skin scars [28, 38]. Limited thoracotomies on both sides of the patient or a mini-sternotomy, possibly in combination with video-assisted techniques, can also be used to encircling ablation of the pulmonary veins [30, 36, 39]. Published data on these new approaches are still limited. The patients involved often concern a highly selected group fulfilling several selection criteria. The reported follow-up is still fairly short. Nevertheless, pulmonary vein isolation has limited results in patients with more complex atrial fibrillation, such as permanent atrial fibrillation and enlarged left atrium [37]. At present it can be concluded that these new approaches to treat atrial fibrillation are feasible, but need to build up evidence to demonstrate efficacy. Obviously, a longer follow up is necessary to be able to draw firm conclusions.

## Results

Taken into account the different surgical strategies, usually the product of evolving techniques, results from different series are not easy to accumulate or to compare. When performed properly, the results obtained from the minimally invasive procedure are good [3]. Although not all series are equally successful, both for isolated as well as concomitant maze procedures results of over 90% sinus rhythm at 6 months after surgery and at midterm follow up are reported, with no additional procedural mortality [14, 40-47].

Taken also into account that surgeons more often treat patients with chronic atrial fibrillation, while cardiologists choose more often to treat patients with paroxysmal atrial fibrillation, differences in the reported success rate in restoring sinus rhythm are a continuing debate among cardiac surgeons and cardiologists on the outcome of maze procedures.

In addition freedom of stroke and freedom of cardiovascular-related death on midterm follow up are reported to be higher as well [47-48].

## Perspective

Maze procedures are not procedures guide by electrophysiological mapping. At least in theory not in all patients all lesions of the maze procedures are necessary. Newer findings have suggested that in selected patients, the atrial arrhythmia may potentially be treated by ablation procedures that are limited to the left atrium or that include only isolation of the pulmonary veins [30, 39]. However, the results of these limited ablations are reported not to be uniformly good [40]. Epicardial mapping systems may be developed that support the understanding of the electrophysiology of the arrhythmia in relation to the clinical manifestation of the patient. Should such systems become a practical tool from the clinical perspective, they may facilitate the surgeon in his surgical strategy to select the appropriate procedure. At present however, mapping patients in atrial fibrillation is complex, and the current intra-operative mapping devices would have to be minimized to allow minimally invasive surgery [49].
